# Chronic Kidney Disease-Mineral and Bone Disorder Management in 4D: The Case for Dynamic Treatment Regime Methods to Optimize Care

**DOI:** 10.1007/s11914-025-00911-8

**Published:** 2025-03-25

**Authors:** Elizabeth Thompson, Adam Tashman, Julia J. Scialla

**Affiliations:** 1 Department of Systems Engineering, School of Engineering, University of Virginia, Charlottesville, VA USA; 2 Department of Medicine, University of Virginia School of Medicine, Charlottesville, VA 800133, USA; 3 School of Data Science, University of Virginia, Charlottesville, VA 22908 USA

**Keywords:** Dynamic treatment regimes, Chronic kidney disease, Dialysis, Secondary hyperparathyroidism, Mineral metabolism, Phosphate

## Abstract

**Purpose of Review:**

Chronic Kidney Disease-Mineral and Bone Disorder (CKD-MBD) is a complex condition impacting patients with kidney failure and characterized by inter-related features such as hyperparathyroidism, hyperphosphatemia, and hypocalcemia. Current treatments include active vitamin D sterols, calcimimetics, and phosphate binders alone and in combination. However, identifying optimal treatment is challenged by interdependency among CKD-MBD features, requiring new approaches to understand dynamic systems. In this review, we discuss challenges and opportunities for a more integrated view of CKD-MBD care.

**Recent Findings:**

Few clinical studies in CKD-MBD care have incorporated a dynamic understanding of the disorder and its treatment. Dynamic treatment regime methods are an evolving area of artificial intelligence (AI) that offer a promising approach for modeling and understanding CKD-MBD care. Efforts to date have included dynamic systems and quantitative systems pharmacology-based models to simulate the impact of alternative treatment regimes. Additional studies utilizing dynamic treatment regime approaches may help improve knowledge gaps in CKD-MBD care.

**Summary:**

Although preliminary research highlights the potential of dynamic treatment regime approaches in optimizing CKD-MBD management, further investigation and clinical validation are necessary to fully harness this approach for improving patient outcomes.

## Introduction

As kidney function declines, progressive abnormalities in calcium and phosphorus homeostasis lead to steady rises in the counter-regulatory hormones, parathyroid hormone (PTH) by the parathyroid gland and fibroblast growth factor 23 (FGF23) from osteocytes in bone [1]. The resulting changes lead to predictable changes in bone structure, including high turnover bone disease and its most severe manifestation of osteitis fibrosis cystica. Recognizing these serious skeletal complications, these changes were traditionally referred to as renal osteodystrophy [2–5]. Over the last quarter century there has been increasing understanding of the myriad effects of these changes in calcium and phosphorus homeostasis, including risk to the cardiovascular system [6, 7]. Young adults on dialysis were found to have diffuse calcification in their coronary arteries, changes otherwise uncommon in this age group [8]. Calcification and stiffening of the large conduit arteries in the cardiovascular system were widely described [9–11]. Taken together changes in mineral (i.e., calcium and phosphorus) homeostasis, hormonal responses, bone, and vascular disease were subsequently defined as a more integrated syndrome affecting nearly all patients with kidney failure on dialysis, known as chronic kidney disease mineral and bone disorder, or CKD-MBD [12].

In this perspective, we argue that the field requires a more sophisticated approach to guide the treatment of CKD-MBD in “4 dimensions (4D)” This includes treatment focused on control of each of the three major CKD-MBD parameters (calcium, phosphorus, and PTH), with an added focus on how they evolve over time and with treatment. Many prior efforts in CKD-MBD have focused on each CKD-MBD parameter in isolation [13], or have considered more integrated phenotypes spanning features, but without an understanding of their dynamic evolution over time [14–17]. A few have evaluated the evolution of CKD-MBD features over time, showing distinct patterns that reflect their interplay[18]. However, these approaches have not yet been able to incorporate time-varying CKD-MBD treatment changes to understand their impact on the natural history of CKD-MBD. Approaches to study CKD-MBD treatments have often considered individual treatments alone [19–22], or have evaluated them as integrated, but static, treatment approaches [23]. As we discuss in this perspective, new approaches using the paradigm of dynamic treatment regimes may help stitch together changes in CKD-MBD features and treatments, and help innovate in the field by better describing and studying fully integrated management of CKD-MBD.

## Chronic Kidney Disease Mineral and Bone Disorder (CKD-MBD) Treatment Options

When presenting with untreated kidney failure, most patients will suffer from secondary hyperparathyroidism, hyperphosphatemia, and often symptomatic hypocalcemia as part of the constellation of CKD-MBD. These core elements have been treated for decades to prevent the most severe disease manifestations.

**Fig. 1:**
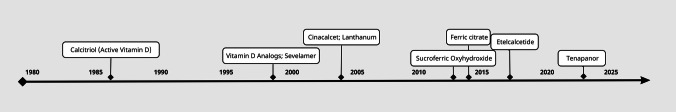
Timeline demonstrating evolving treatment options in chronic kidney disease mineral and bone disorder

The mainstay of treatment for secondary hyperparathyroidism and hypocalcemia since the 1970s has been active vitamin D sterols (Fig. 1). Use of active vitamin D best addresses a core pathophysiologic feature of CKD-MBD, which centers on reduced endogenous production of calcitriol, and also helps maintain normo-calcemia. Vitamin D analogs are synthetically modified versions of calcitriol that reduce PTH but have a more modest impact on raising calcium [24–29]. These were introduced in the 1990s, and have been associated with better outcomes in observational studies [28, 30, 31]. Active vitamin D sterols, either calcitriol or its analogs, are used by approximately 80% of patients with kidney failure on hemodialysis [32].

Calcimimetics are alternatives or adjuncts to active vitamin D sterols to lower PTH. Calcimimetics lower PTH by altering the sensitivity of the calcium-sensing receptor on the parathyroid gland causing a lower calcium threshold to stimulate PTH release [33]. The first-in-class calcimimetic, cinacalcet, was approved by the US Food and Drug Administration in 2004 [33]. An intravenous calcimimetic, etelcalcetide, was approved in the US in 2017 [34, 35]. Calcimimetics are used by approximately 30% of patients on hemodialysis in the US [32].

Typically, three-times per week dialysis does not remove enough phosphorus to maintain neutral balance and thus gastrointestinal phosphate binders are typically needed to prevent hyperphosphatemia and deposition of phosphate in the skin and vasculature [36]. At the inception of dialysis, calcium carbonate, magnesium- or aluminum-containing binders were often used. In the 1990s, alternate calcium-containing binders (e.g., calcium acetate) and non-calcium based resins, such as sevelamer, became available. In the 2000s, metal-containing binders, such as lanthanum carbonate were introduced. The newest treatments for hyperphosphatemia include iron-containing binders (e.g., succroferric oxyhydroxide; ferric citrate), and a novel agent, tenapanor, that was approved by the US Food and Drug Administration in 2023 to limit gastrointestinal absorption through paracellular transport in the intestine [37]. Altogether > 80% of patients who are treated with hemodialysis in the US use gastrointestinal phosphate binders [32, 36].

## CKD-MBD as a Case Study in Dynamic Treatment Regimes

As discussed above, a variety of agents are available to treat CKD-MBD, each with relatively rapid effects and biologic interactions [38]. For instance, calcimimetics lower PTH while also lowering calcium and phosphorus [33, 34], whereas active vitamin D sterols lower PTH while raising calcium and phosphorus [39]. Phosphate binders lower phosphorus and may have neutral effects on calcium, or may raise calcium depending upon the type of binder chosen [36]. In combination, addition of active vitamin D sterols to a regimen including a calcimimetic may help ameliorate hypocalcemia caused by the calcimimetic alone, while providing additional PTH reduction [40].

**Fig. 2:**
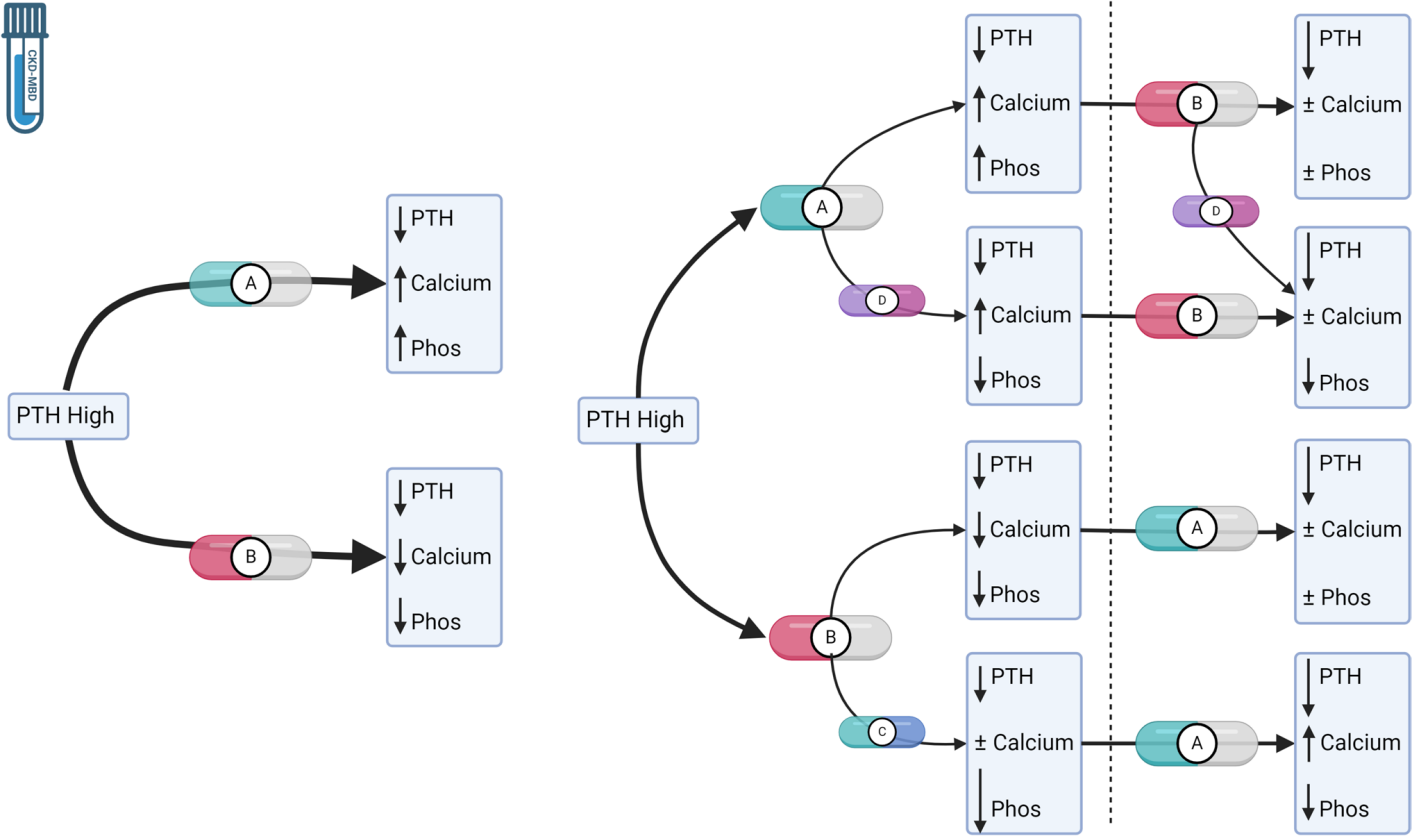
Schematic Illustration of Dynamic Treatment Regimes in Chronic Kidney Disease Mineral and Bone Disorder (CKD-MBD). In this depiction, treatment is focused on correction of secondary hyperparathyroidism, a common component of CKD-MBD in which parathyroid hormone (PTH) is severely elevated because of the abnormal internal milieu in kidney failure. Secondary hyperparathyroidism can be treated with one of two first line classes of medications as depicted in the simplified far left panel. Active vitamin D sterols, depicted here as medication “A”, can lower PTH while also raising serum calcium and phosphorus (phos) primarily by increasing gastrointestinal absorption. Alternatively, secondary hyperparathyroidism can be treated with calcimimetics, which are agonists of the calcium-sensing receptor present on the parathyroid gland and other organs. Calcimimetics are pictured as medication “B” and can lower PTH while tending to decrease serum calcium and phos. These contrasting ‘off-target’ effects on serum calcium and phos create the major dose-limiting effects of these drugs, but they can be ameliorated by use in combination or with additional co-interventions as we depict in the more complex panel on the right. For instance, to mitigate impacts of active vitamin D sterols (medication “A”) or calcimimetics (medication “B”) on serum calcium or phos, gastrointestinal phosphate binders may be added. Here, in the first time-step, a non-calcium containing phosphate binder (medication “D”) may be added to active vitamin D sterol to control serum phos. A calcium-containing phosphate binder (medication “C”) may be added to a calcimimetic to help normalize serum calcium and further lower phos. In the second depicted time-step (after the dashed line), further steps may be taken to normalize serum calcium and phos and improve PTH control. For instance, a calcimimetic (medication “B”) may be added to the active vitamin D sterol-based treatment with or without a phosphate binder. In a calcimimetic-based treatment regime, active vitamin D sterols may be added to counteract effects on serum calcium and phos. These are examples depicting the dynamic, iterative treatment adjustment that is common in hemodialysis to maintain control of CKD-MBD. Additional complexity could include changes in dialysate calcium or extension of hemodialysis treatment time, which will lower serum phos. Created with BioRender.com

Typically, these CKD-MBD agents act within days to weeks [30, 41, 42], and thus frequent monitoring and adjustment are a core part of management. In most current hemodialysis practices calcium and phosphorus are measured monthly and PTH is measured quarterly, often with more measurements needed if medication changes are made [32, 43]. The evolving state (e.g., CKD-MBD laboratories) of the patient is considered after each measurement, resulting in medication adjustment in a dynamic treatment regime [32, 44]. A schematic description of the complex interplay of medications and the evolving laboratory state of patients in CKD-MBD care is depicted in Fig. 2.

## Limitations of Prior Studies in CKD-MBD

Despite years of investigation, the current best practices for CKD-MBD care in dialysis are uncertain. Although a variety of treatment trials have assessed the effectiveness of different medications and classes, most of these trials have primarily focused on single agents, rather than treatment strategies that employ agents in combination [38]. Where they have defined combination approaches, they have typically focused only on short term biochemical outcomes [45–47]. A few clinical trials have studied important clinical outcomes, randomizing patients to different treatments over longer time horizons [48–50]. Unfortunately, many of these studies have been plagued by high degrees of cross-over and drop out yielding inconclusive results [48, 49]. Simplified treatment approaches randomizing to a single drug versus placebo over time are not able to fully capture the integrated complexity of CKD-MBD care and cannot describe the dynamic titration employed in practice, during which care may need to change as CKD-MBD evolves or due to biochemical changes induced by prior treatment titrations.

Our team has tried to address some of these challenges by assessing the impact of different CKD-MBD treatment ‘actions’ rather than static parameters, such as the level of calcium, phosphorus or PTH, or use of a specific medication [51]. Using an observational clinical trial emulation, we evaluated the impact of titration of CKD-MBD agents within a specific, clinically relevant context, similar to the anticipated inclusion criteria of an analogous clinical trial. We identified patients on hemodialysis at the time of their first PTH between 300–600 pg/ml who had not had a recent CKD-MBD medication titration. If we observed an upward titration of a CKD-MBD medication within 30 days of this result, we classified this patient as being treated with a ‘lower PTH target’ approach versus a patient whose clinician did not titrate in this interval. Our analysis found improved cardiovascular and mortality outcomes in patients whose clinicians were more proactive in titrating, presumably in pursuit of the lower PTH target [51]. This analysis has the advantage of studying a dynamic aspect of the provider’s treatment approach (i.e., medication titration, as opposed to only which medication was chosen or which biochemical parameter was achieved). However, it remains limited in being able to describe only one aspect of care (e.g., the PTH target), and focusing on an initial titration without being able to capture subsequent downstream biochemical changes and subsequent titrations. In order to fully define and study different treatment approaches that may evolve over time, new approaches are needed for the problem of CKD-MBD using the dynamic treatment regime framework.

## Artificial Intelligence and Other Tools to Study Dynamic Treatment Regimes

A variety of Artificial Intelligence (AI) techniques are available to model dynamic treatment regimes and potentially guide medication decisions [52]. Examples in the literature include anemia management, sepsis, glycemic management in diabetes, anti-coagulation dosing, and CKD-MBD [53–61]. A dynamic treatment regime can be thought of most simply as an iterative decision-making process, whereby actions taken in a current state may impact the patient’s next state, and so on. The care provider must decide which action to take at each time step to achieve the best outcome, a common scenario in CKD-MBD care.

Several approaches have been explored for developing algorithms for dynamic treatment regimes, including dynamic systems models and reinforcement learning [62]. Dynamic systems models are numerical simulations developed to mimic the problem space, allowing for an optimal solution to be simulated. They have had limited implementation in the healthcare field to date [63, 64]. A challenge in developing improved solutions for dynamic treatment regimes is the (very often) lack of a correct answer, or *ground truth*, associated with the data. A dataset without the ground truth values is called an *unlabeled dataset*.

Reinforcement learning has shown significant success in optimization problems with unlabeled data [62, 65]. The structure of reinforcement learning is based on the interactions between an agent (in this case the patient) and the environment. The approach uses time series data set up as a series of states, actions, rewards, and next states to learn an optimal policy based on a provided value system. Given any possible patient state, the policy will provide the recommended action that is estimated to provide the greatest long-term value to the agent. Value estimates are calculated based on an explicit reward function, which assigns rewards and penalties based on the next state following the action. In CKD-MBD, a reward function could theoretically be designed for simultaneous control of PTH, calcium, and phosphorus to certain targets. Instead of a simple rules-based treatment algorithm focused exclusively on selecting treatment based on the patient’s current state (e.g., CKD-MBD laboratories), the approach can include covariate and past state information (i.e., environment). Reinforcement learning can also obtain optimal policies balancing immediate (e.g., expected parameters next month) and future rewards (e.g., expected parameters in several months). Several forms of reinforcement learning have been used to develop and optimize dynamic treatment regimes including Q-learning, policy gradient methods, and deep reinforcement learning [52]. These and related AI approaches are ideally suited to the dynamic treatment regime problem encountered in CKD-MBD care.

## Applications of AI in CKD-MBD

The progress in utilizing AI to optimize dynamic treatment regimes differs greatly across medical conditions. For CKD-MBD treatment, use of dynamic treatment regimes is still in its early stages although a few prior examples are published.

Schappacher-Tilp et al. [66] developed a mathematical model of parathyroid gland biochemical processes that was centered around the calcium-sensing receptors (CaSR) in parathyroid gland cells. This model can then be used to test the impact of medication combinations, such as calcimimetics and active vitamin D sterols, by incorporating the mathematical models for those medication effects into the CaSR model. Additionally, as long as there are no overlapping input parameters, this model can be combined with other models, for example a bone model. This allows for further analysis regarding interactions between the two physiological systems, and the impact of various medical treatment combinations on the combined system. Application of this model in 26 patients with kidney failure on hemodialysis demonstrated initial promising results about the accuracy of the model in anticipating immediate peri-dialytic changes in PTH after calcium loading, and longer-term changes in PTH based on calcium and phosphorus trajectories in 13 patients [67].

Gaweda, Brier and Lederer [68] developed a quantitative systems pharmacology (QSP) model of CKD-MBD by modifying the Peterson and Riggs QSP models of calcium and phosphorus metabolism to account for physiological differences inherent in individuals struggling with CKD-MBD. They incorporated additional relevant parameters, like the phosphorus-regulatory hormone FGF23, and altered several equations to better simulate the physiology of individuals with kidney failure, including equations related to the parathyroid gland and phosphorus regulation. They validated the model fit using historical clinical data, and demonstrated that the new modified model had significantly lower error metrics when compared with the original model. In 2022 Gaweda, Brier and Lederer [54] expanded on their original QSP approach by incorporating deep reinforcement learning. The expanded model includes the addition of three medication classes used to treat CKD-MBD: calcimimetics, active vitamin D sterols, and phosphate binders. The reinforcement learning component of this expanded methodology uses the QSP model to represent the environment, then calculates optimal treatment policy using dosage changes in medication classes as the available actions, with the state space consisting of calcium, phosphorus and PTH biochemical parameters. Using simulated data, researchers compared the performance of the reinforcement learning trained AI in comparison with an AI trained using behavioral cloning (BC), a method that mimics the human decision-making process. They found that the reinforcement learning agent was able to achieve and maintain a desired state quicker and more accurately than the BC agent trained on the simulated human expert dataset. They also noted from the simulation that the reinforcement learning agent used phosphate binders and calcimimetics less frequently than the BC agent, and it used active vitamin D sterols more frequently. This suggests that shifting medication practices towards favoring active vitamin D sterols could improve overall treatment prognosis for CKD-MBD. More recently, they have used their QSP and RL approach to simulate different approaches to treating CKD-MBD, including approaches focused on achieving current guidelines targets versus preventing certain unfavorable pathophysiologic processes (e.g., calcium efflux from bone) [69]. This approach could be used to generate new hypotheses about how to treat CKD-MBD and predict downstream actions of new treatments [70, 71].

Future steps are needed to validate these promising systems models in empiric human data and to assess their accuracy and utility prospectively. Other empirical approaches that are less reliant on proper simulated model specification and instead use observed clinical data to model dynamic treatment regimes for CKD-MBD should also be developed.

## Conclusion

CKD-MBD is characterized by biological interactions, multiple treatment options, and iterative biological feedback on the system, providing an ideal setting to standardize and improve care through the structured paradigm of dynamic treatment regimes. AI approaches to optimize care in dynamic treatment regimes are well poised to substantially innovate in this space, providing new insight into optimal treatments based on systems biology insights or other reinforcement learning approaches. Work is needed to test these approaches to CKD-MBD care in trials and improve management for patients with kidney failure.

## Key References

- Neri L, Kreuzberg U, Bellocchio F, Brancaccio D, Barbieri C, Canaud B, et al. Detecting high-risk chronic kidney disease-mineral bone disorder phenotypes among patients on dialysis: a historical cohort study. Nephrol Dial Transplant. 2019;34(4):682-91.
  - Using electronic health record data in over 35,000 patients with kidney failure on dialysis internationally, the investigators assessed the association of different CKD-MBD phenotypes with clinical outcomes over 5 years. The CKD-MBD phenotypes included all potential combinations of calcium, phosphorus and PTH: low, target, or high. The highest risk phenotypes for mortality and hospitalization tended to have more than one CKD-MBD abnormality, suggesting that the features should not be assessed in isolation.
- Filipozzi P, Ayav C, Ngueyon Sime W, Laurain E, Kessler M, Brunaud L, et al. Trajectories of CKD-MBD biochemical parameters over a 2-year period following diagnosis of secondary hyperparathyroidism: a pharmacoepidemiological study. BMJ Open. 2017;7(3):e011482.
  - Among a cohort of 269 patients with secondary hyperparathyroidism on dialysis, investigators defined trajectories of CKD-MBD laboratories over approximately 2 years, including calcium, phosphorus and PTH. They identified 4 groups characterized as 'rapid PTH drop', 'gradual PTH decrease', 'slow PTH decrease with high phosphate', and 'uncontrolled secondary hyperparathyroidism'. This is an example of a study assessing the evolution CKD-MBD parameters over time although changes in treatments could not be incorporated.
- Karaboyas A, Muenz D, Fuller DS, Desai P, Lin T-C, Robinson BM, et al. Etelcalcetide Utilization, Dosing Titration, and Chronic Kidney Disease-Mineral and Bone Disease (CKD-MBD) Marker Responses in US Hemodialysis Patients. Am J Kidney Dis. 2022;79(3):362-73.
  - This study evaluated over 2,000 patients on maintenance hemodialysis to assess changes in CKD-MBD laboratory trajectories after new use of etelcalcetide. The laboratory trajectories demonstrated improvement in PTH, as well as lowering of calcium and phosphorus. They also noted increased use of co-interventions such as active vitamin D sterols, calcium-containing phosphorus binders and higher dialysate calcium, demonstrating the integrated and dynamic nature of CKD-MBD management even in studies focused on single agents.
- Platt A, Wilson J, Hall R, Ephraim PL, Morton S, Shafi T, et al. Comparative Effectiveness of Alternative Treatment Approaches to Secondary Hyperparathyroidism in Patients Receiving Maintenance Hemodialysis: An Observational Trial Emulation. Am J Kidney Dis. 2024;83(1):58–70.
  - In this study, the investigators utilized electronic health record data from patients with kidney failure on in-center hemodialysis with new onset secondary hyperparathyroidism to conduct a clinical trial emulation. Evaluating medication titration patterns in response to first PTH 300–600 pg/ml, the investigators determined that patients who had more proactive treatment (e.g., upward titration of CKD-MBD medications) within 30 days of first PTH 300–600 pg/ml had lower rates of mortality and cardiovascular disease over 2 years. This is an observational study but suggests the need for a clinical trial of lower PTH treatment targets in CKD-MBD.
- Gaweda AE, Lederer ED, Brier ME. Artificial intelligence-guided precision treatment of chronic kidney disease-mineral bone disorder. CPT Pharmacometrics Syst Pharmacol. 2022;11(10):1305-15.
  - In this simulation, the authors utilized a quantitative systems pharmacology model that captures the complexity of the biological systems involved in CKD-MBD treatment. They then used Reinforcement Learning (RL) with rewards aligned with clinical practice guidelines. Using synthetic subjects, the authors demonstrated how RL can create policy recommendations for CKD-MBD care that are distinct from an a priori rules-based approach.

## Data Availability

No datasets were generated or analysed during the current study.
